# Effectiveness of home-based exercise interventions on pain, physical function and quality of life in individuals with knee osteoarthritis: a systematic review and meta-analysis

**DOI:** 10.1186/s13018-023-04004-z

**Published:** 2023-07-17

**Authors:** Juncheng Si, Lili Sun, Zheng Li, Wenning Zhu, Weidong Yin, Lina Peng

**Affiliations:** Harbin Sport University, Harbin, China

**Keywords:** Home-based exercise, Knee osteoarthritis, Pain, Physical function, Quality of life

## Abstract

**Objective:**

The objective of the study was to evaluate the effectiveness of home-based exercise interventions on pain, physical function and quality of life in individuals with knee osteoarthritis (KOA).

**Methods:**

Five databases (PubMed, Embase, Cochrane Library, CINAHL, Web of Science Core Collection) were searched for relevant randomized controlled trials (RCTs) published from database inception to 2 August 2022. The Cochrane Collaboration’s standards were followed for study selection, eligibility criteria, data extraction and statistics, using the Cochrane Collaboration Risk of Bias Tool and PEDro for quality assessment. A meta-analysis and subgroup analyses, stratified by control condition and intervention duration, were conducted using RevMan 5.4. The study was reported in compliance with the PRISMA statement.

**Results:**

A total of 12 independent RCTs with 1442 participants were included. The meta-analysis showed that the home-based exercise interventions significantly reduced pain in individuals with KOA (SMD =  − 0.32, 95% CI [− 0.41, − 0.22], *p* < .01) and improved physical function (SMD =  − 0.25, 95% CI [− 0.47, − 0.02], *p* = .03) and quality of life (SMD = 0.63, 95% CI [0.41, 0.85], *p* < .001). Subgroup analysis revealed that home-based exercise interventions were superior to health education and no treatment, in terms of pain and physical function, and similar to clinic-based exercise and pharmacologic treatment.

**Conclusions:**

The effect of home-based exercise intervention is significantly better than health education and no treatment for reducing knee pain and improving physical function, and was able to achieve the effects of clinic-based exercise treatment and pharmacologic treatment. With regard to quality of life, the unsupervised home strength exercise intervention showed a significant effect compared with the health education control and combined with cognitive behavioural therapies may produce better results. Although home-based intervention provides effective treatment options for individuals with clinical treatment limitations, individual disease complications and the dosimetry of exercise need to be considered in practice. Furthermore, growing evidence supports the effectiveness of Tai Chi in the rehabilitation of KOA.

**Supplementary Information:**

The online version contains supplementary material available at 10.1186/s13018-023-04004-z.

## Introduction

Osteoarthritis is a highly prevalent whole-joint disease and the global number of people affected has increased by 48% from 1990 to 2019 [1]. Knee osteoarthritis (KOA), being the most common [2], accounted for approximately 85% of the burden of osteoarthritis worldwide as early as 2016 [3]. Depending on the source, roughly 13% of women and 10% of men aged 60 years and older have symptomatic KOA. Among those older than 70 years of age, the prevalence rises to as high as 40% [4]. The majority of KOA individuals experience pain. Severe pain can cause physical dysfunction and lower their quality of life [5, 6]. There is no cure for KOA and total knee arthroplasty is the only reliable option for the individuals with severe KOA [7] which may have a significant impact on the healthcare system and family economic costs.

Recommendations for KOA treatment are often separated into non-pharmacological, pharmacological and surgical interventions. Long-term medication can increase the risk of adverse events (e.g. gastrointestinal and cardiovascular events) and most surgical treatments have known risks [8]. Exercise is a core non-pharmacological intervention that has been recommended by the European Society for Clinical and Economic Aspects of Osteoporosis (ESCEO) and Osteoarthritis Research Society International (OARSI) [9]. However, exercise conducted in the clinic may be limited, such as during the coronavirus disease 2019 (COVID-19) pandemic [10]. A growing body of research suggests that home-based exercise intervention (HBEI) appears to be a more preferable form of intervention [11, 12]. As a crucial complement to outpatient rehabilitation therapy, HBEI reduces clinic visits, clinic waiting time and the costs incurred from transportation to the clinic, while also offering a high level of treatment [13], and may be a suitable and preferred choice for individuals suffering from KOA who are unable to go to the clinic for help [12].

To our knowledge, there is only one meta-analysis focusing on KOA management, which found that home exercise programmes could improve pain and function, but it did not focus on individuals' quality of life [11]. In addition, there is only one review focusing on management of KOA before surgery, but there may be a risk of bias [12]. Recently, a number of new original studies on the effect of HBEI in individuals with KOA have been conducted and mixed results have been reported. Therefore, these new studies were included in this meta-analysis with the aim of exploring the impact of HBEI on pain, physical function and quality of life in individuals with KOA.

## Methods

This systematic review and meta-analysis was conducted according to the Preferred Reporting Items for Systematic Reviews and Meta-Analyses (PRISMA) guidelines [14]. The study protocol was registered in PROSPERO (No.: CRD42022350513).

### Search strategy

The search strategy was carried out based on the components of population, intervention, comparison, outcome and study design (PICOS) and consisted of free text words and Medical Subject Heading (MeSH) terms, including “home”, “knee osteoarthritis”, “exercises”, “physical activity” and “randomized controlled trial”. Original articles were searched in PubMed, Embase, Cochrane Library, CINAHL and the Web of Science Core Collection from database inception to 2 August 2022. The full search strategies are available in Additional file 1: Appendix.

### Criteria for selection of studies

The trials selected in this review met the following inclusion criteria: (1) randomized controlled trials (RCTs) written in English; (2) participants aged 40 years or older who were diagnosed with KOA by a physician according to American College of Rheumatology (ACR) clinical criteria for KOA or based on radiographic evidence or local clinical criteria for KOA; and (3) pain, physical function or quality of life as one of the outcome measures.

The following were the criteria for exclusion: (1) individuals who had undergone knee arthroplasty or were waiting for surgical interventions, or who had mental illness, neurological conditions or terminal illness; (2) studies that include home exercise programmes in their control group; and (3) interventions that did not include home exercise programmes.

### Interventions and controls

Home-based exercise was defined as any exercise that occurs in the home (e.g. strengthening exercise, flexibility training, balance training or traditional Chinese sports) that can be combined with other interventions. The exercise programme is implemented via traditional or electronic technology-related delivery (e.g. exercise booklet, telephone and web/smartphone applications). In addition, interventions could be supervised exercise or unsupervised individual exercise; the supervision and guidance of physiotherapists can be achieved through traditional face-to-face exercise treatment or a variety of telecommunication tools.

The control groups received programmes that consisted of exercise treatment in the clinic (individual or group), no intervention (no specific intervention or received a placebo), health education (e.g. booklet, lectures, leaflet, internet-based material) or pharmacologic treatment (taking non-steroidal anti-inflammatory drugs or injected hyaluronate). Because most participants may not be able to self-inject hyaluronate, two situations were considered in this review: (1) the therapist injects the participant at home; and (2) the participant goes to the clinic for injection.

### Outcomes

The primary outcomes were pain, which could be measured by the Western Ontario and McMaster Universities Osteoarthritis Index (WOMAC) pain subscale, the Visual Analogue Scale (VAS), the Numerical Rating Scale (NRS), etc., and physical function, which could be measured by the WOMAC functional subscale, the Japanese Knee Osteoarthritis Measure (JKOM), the Ibadan Knee/Hip Osteoarthritis Outcome Measure (IKHOAM), etc. The second outcome is quality of life, which could be measured by the Medical Outcomes Survey Short Form (SF-36), the Assessment of Quality of Life (AQoL), the Arthritis Impact Measurement Scales 2 Short Form (AIMS2-SF), etc. The most representative scale was selected for analysis if multiple scales were used to evaluate the same outcome in a study.

### Selection process

After de-duplication of all retrieved literature by Endnote X9 and manual screening, two trained reviewers independently screened the titles, abstracts and then the full texts to select potentially eligible literature strictly according to the inclusion and exclusion criteria. Any discrepancies between the two reviewers were resolved by a third reviewer.

### Data collection

Data were extracted independently by two trained reviewers using a standard form. The extracted data included the following information:

- *Publication details*: Authors, publication year, country.
- *Participants*: Sample size, mean age of participants, percentage of female participants, body mass index (BMI).
- *Home-based exercise interventions*: Content, frequency, duration.
- *Outcomes*: Mean difference and standard deviation (SD) within groups for pain, physical function and quality of life.

The author was contacted if the mean change and SD of an outcome could not be found in the article or calculated from the available data.

### Assessment of methodological quality

The RCTs were evaluated using the Cochrane Collaboration Risk of Bias Tool, which assesses random sequence generation, allocation concealment, blinding of participants and personnel, blinding of outcome assessment, incomplete outcome data and selective reporting and other bias. In addition, the PEDro scale was used to assess the methodological quality of the included studies. Two reviewers assessed each item independently and any discrepancies were resolved by a third reviewer.

### Statistical analysis

RevMan 5.4.1 was used to conduct the meta-analysis. Because the outcomes were continuous variables, the mean difference and standard deviation between baseline and post-intervention within groups were used to calculate the total effect size. A fixed-effect model was used when no significant heterogeneity was observed (*p* > 0.05 and I^2^ < 50%); otherwise, a random-effect model was applied. Forest plots were used to present the pooled estimate. A funnel plot and Egger's test were used to assess publication bias using Stata/PM (version 17.0). When meta-analysis could not be performed, the results were presented in narrative form. In addition, subgroup analyses were conducted according to the type of treatment received by the control groups.

## Results

### Search outcome

**Fig. 1:**
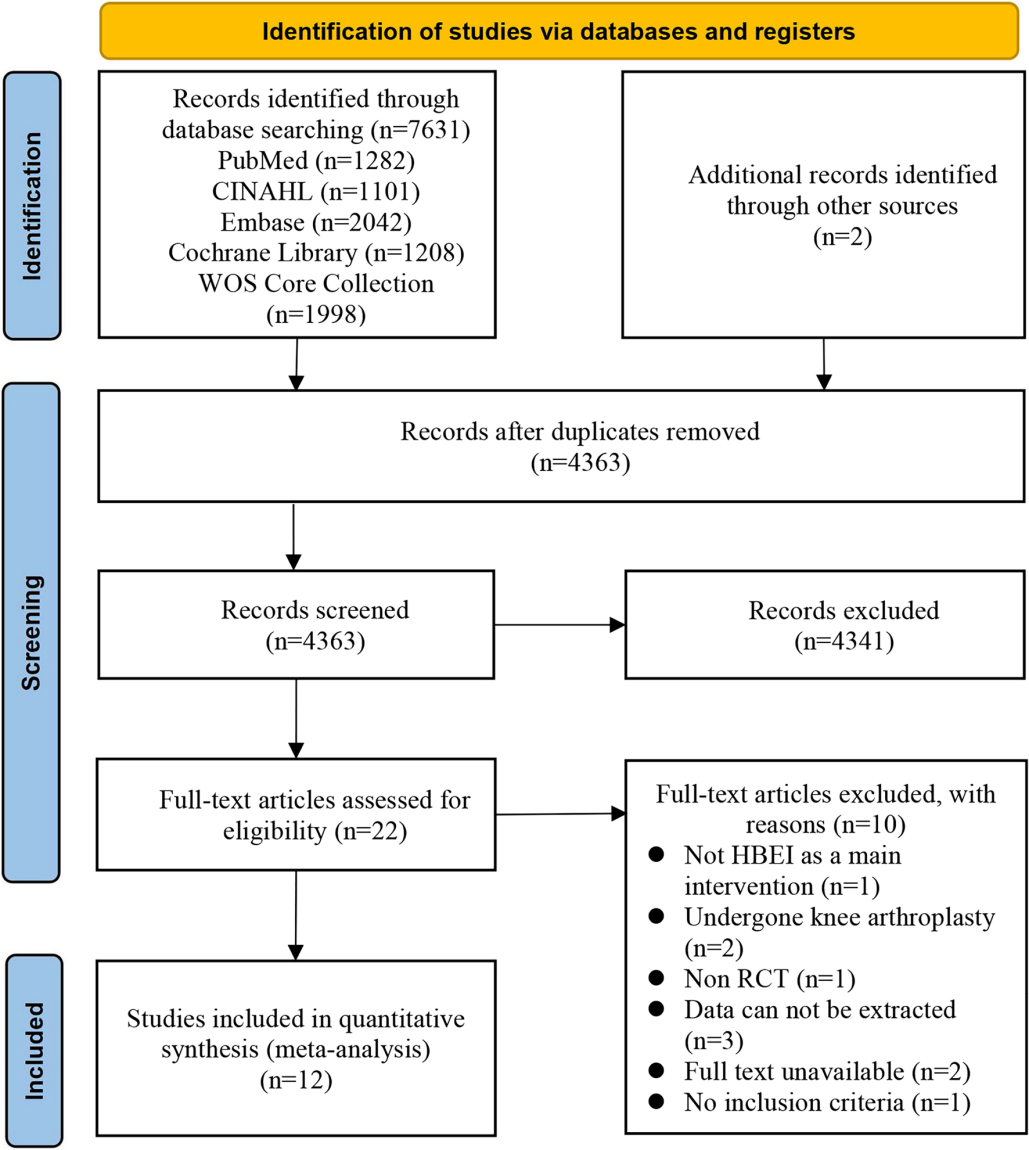
PRISMA flowchart presenting the summary of searches carried out in the literature. HBEI indicates home-based exercise intervention; RCT, randomized controlled trial

A total of 7631 records were retrieved from the databases and reference lists, of which 3268 duplicate records were removed by Endnote X9 and 4341 irrelevant records were excluded by reading the titles and abstracts. Therefore, 22 records were screened for full text and 10 studies were further excluded for the following reasons: the contrast of one study was not HBEI as a main intervention; the populations of two studies had undergone knee arthroplasty; the approach of one study was not an RCT; the necessary data of three studies could not be extracted; and the full texts of two studies were not available. In addition, the diagnostic criteria of one study were not reported. Therefore, 12 independent RCTs were included in the final analysis of this review, details of which are shown in Fig. 1.

### Characteristics of the included studies

The 12 independent RCTs comprised 1442 participants in total: three studies (25%) were from the United States [15–17], three (25%) from the United Kingdom [18–20], two (17%) from Japan [21, 22] and and the other four (33%) from China [23], Nigeria [24], Australia [25] Turkey [26]. The sample sizes of included studies were between 33 and 313. The mean age of participants with KOA ranged from 56.04 to 71.2 years. Across all 12 studies the percentage of females ranged from 44.0% to 100.0% and 10 studies reported the average BMI of participants, which ranged from 24.5 to 34.8.

Of the 12 studies identified, with regard to the control conditions, two studies (17%) received a clinic-based exercise treatment [24, 26], no interventions in three RCTs (25%) [16, 18, 19], health education in five RCTs (41%) [15, 17, 20, 23, 25], and pharmacologic treatment in the other two RCTs (17%) [21, 22]. As for home exercise intervention contents, one study (8%) consisted of standardized home-exercise programmes and no detailed exercise plan was reported [24], 10 RCTs (84%) used strengthening exercises, or combined with kinesthesia, balance and agility exercise [15, 16, 18–23, 25, 26], and Tai Chi was used in one RCT (8%) [17]. The detailed characteristics of the included studies are shown in Table 1.

**Table 1:** Characteristics of the included studies

Authors/ Country	Participants	Number (n) and age [mean (SD)] of participants	BMI [mean (SD)]	Female (n%)	HBEI contents	Control group	Outcome	Effects founds
O'Reilly (1999)^18^ The United Kingdom	Diagnostic: ACR Inclusion: knee pain, age: 40–80 years	HBEI: n = 113 (61.94 ± 10.01) CON: n = 78 (62.15 ± 9.73)	–	HBEI: 64% CON: 68%	Strengthening exercise program for lower-limb muscles, a maximum of 20 repetitions on each leg a daily for 6 months	Not intervention	Pain: WOMAC, VAS PF: WOMAC	Pain scores were reduced by 22.5% in the home exercise group and by 6.2% in the control group WOMAC score was reduced by 17.4% in the home exercise group and was unchanged in the control group
Baker (2001)^15^ USA	Diagnostic: x-ray Inclusion: aged > 55 years, Body mass index ≤ 40 kg/m2, pain on more than half the days of previous month during activities	HBEI: n = 23 (69.0 ± 6.0) CON: n = 22 (68.0 ± 6.0)	HBEI: 31 ± 4.0 CON: 32 ± 5.0	HBEI: 74% CON: 86%	Home based progressive strength, performed 2 sets of 12 repetitions, 3 times per week for 4 months	Nutrition education booklet	Pain: WOMAC PF: WOMAC QoL: SF-36	Home-based progressive strength training program significantly reduces pain, improves physical function by approximately 30% greater and quality of life than control group
Thomas (2002)^19^ The United Kingdom	Diagnostic: x-ray Inclusion: knee pain, age: > 45 years	HBEI: n = 235 (61.5 ± 9.58) CON: n = 78 (61.9 ± 9.39)	HBEI: 28.02 ± 4.18 CON: 28.14 ± 4.81	–	Graded elastic bands were used to do resistance training, perform the programme with both legs for 20–30 min a day, increase the number of repetitions up to a maximum of 20 per leg for 24 months	Placebo health food tablet	Pain: WOMAC	Home-based exercise programmes can produce significant reductions in knee pain over two years
Brismee (2007)^17^ USA	Diagnostic: American Rheumatism Association Inclusion: aged 50 years or older with knee pain	HBEI: n = 22 (70.89 ± 9.8) CON: n = 19 (68.89 ± 8.9)	HBEI: 27.96 ± 5.92 CON: 27.70 ± 6.57	HBEI: 86% CON: 78%	The 24-form simplified Yang-style Tai Chi, three times a week for 6 weeks	Health lectures	Pain: VAS PF: WOMAC	Six weeks of home-based Tai Chi may provide knee pain reduction and physical function improvement The positive effects of tai chi was not sustained after six weeks of detraining
Doi (2008)^21^ Japan	Diagnostic: American Rheumatism Association Inclusion: knee pain, age ≥ 50 years, osteophytes confirmed by x-rays	HBEI: n = 72 (66.8 ± 12.8) CON: n = 70 (68.9 ± 21.1)	HBEI: 24.8 ± 3.5 CON: 24.5 ± 3.8	HBEI: 76% CON: 76%	Exercise the quadriceps muscle group by performing knee extension movements, performed four sets of 20 repetitions of the above quadriceps exercise every day for 8 weeks	Taking the nonsteroidal anti-inflammatory drugs (NSAIDs)	Pain: VAS PF: WOMAC, JKOM	Both quadriceps strengthening exercise and NSAIDs significantly decreased knee pain and improved daily activity and social participation
Jenkinson (2009)^20^ The United Kingdom	Diagnostic: x-ray Inclusion: BMI ≥ 28, aged 45 years or older with knee pain	HBEI: n = 82 (61.1 ± 9.8) CON: n = 76 (61.5 ± 9.2)	HBEI: 34.8 ± 6.6 CON: 33.0 ± 6.5	HBEI: 68% CON: 65%	Unsupervised home-based quadriceps strengthening exercise, complete ≥ 2 exercises a day, with 5 to 20 repetitions of each exercise for 24 months	Advice leaflet	Pain: WOMAC	A home based, simple knee strengthening exercises over a two year period can significantly reduce knee pain
Kawasaki (2009)^22^ Japan	Diagnostic: ACR Inclusion: Postmenopausal female (> 50 years of age) with primary OA of the medial femorotibial compartment	HBEI: n = 52 (71.2 ± 7.1) CON: n = 50 (69.5 ± 8.4)	HBEI: 24.6 ± 3.0 CON: 25.7 ± 4.1	HBEI: 100% CON: 100%	Isometric muscle exercises of the bilateral lower limbs, twice a day for 24 weeks	The intraarticular injection of hyaluronate (HA) group	Pain: VAS PF: JKOM	Both hyaluronate injections and exercise equally resulted in relief of pain and functional improvement
Rogers (2012)^16^ USA	Diagnostic: ACR Inclusion: self-reported knee pain, minimum disability score of 17 points on physical function subscale of WOMAC. Aged 50 yeas and older	HBEI (KBA): n = 8 (70.7 ± 10.7) HBEI (RT): n = 8 (70.8 ± 6.5) HBEI (KBA + RT): n = 9 (68.8 ± 10.1) CON: n = 8 (71.2 ± 10.9)	HBEI (KBA): 28.9 HBEI (RT): 28.2 HBEI (KBA + RT): 29.2 CON: 30.8	HBEI (KBA): 69% HBEI (RT): 70% HBEI (KBA + RT): 75% CON: 67%	KBA: utilized walking agility exercises plus single-leg static and dynamic balancing RT: use non-latex elastic resistance bands to perform a single 15-repetition set of lower extremity exercises with each leg KBA + RT: participants performed selected exercises from each technique Three times per week for 30–40 min for 8 weeks	Applied inert lotion daily	Pain: WOMAC PF: WOMAC	KBA, RT, or a combination of the two administered as home exercise programs appear effective in reducing symptoms and improving physical Function
Odole (2013)^24^ Nigeria	Diagnostic: out-patient physiotherapy clinics In three health care facilities in Nigeria Inclusion: diagnosis of OA of the knee joint; literacy in English or Yoruba language and the means to communicate via mobile telephone	HBEI: n = 25 (56.04 ± 7.40) CON: n = 25 (54.96 ± 7.81)	–	HBEI: 44% CON: 52%	Physiotherapists monitored and coached patients via the mobile telephone guide,3 times per week for 6-weeks	Receiving the same standardized exercise in the clinic	Pain: VAS PF: IKHOAM	There were significant improvement in pain and physical function in patients with osteoarthritis of the knee following 6-week of tele-physiotherapy intervention
Bennell (2017)^25^ Australia	Diagnostic: NRS, WOMAC Inclusion: aged 50 years or older, knee pain for more than 3 months and on most days of the previous month	HBEI: n = 74 (60.8 ± 6.5) CON: n = 74 (61.5 ± 7.6)	HBEI: 32.0 ± 13.9 CON: 30.1 ± 10.2	HBEI: 58% CON: 54%	Internet-based home exercise, included educational material and a lower-limb–strengthening exercise program, 3 times per week for 9 months	Internet-based educational material	Pain: NRS, WOMAC PF: WOMAC QoL: AQoL-2	Internet-delivered, physiotherapist-prescribed exercise provide clinically meaningful improvements in pain and function that are sustained for at least 6 months
Colak (2017)^26^ Turkey	Diagnostic: clinically and radiographically Inclusion: age 45 years or older, Kellgren–Lawrence Grade II–III OA determined clinically and radiographically	HBEI: n = 39 (59) CON: n = 39 (60)	HBEI: 30.08 ± 4.35 CON: 31.82 ± 6.49	HBEI: 65% CON: 73%	Lower extremities muscle strengthening exercise and simple balance exercises, 40–45 min a day, 3 times per week for 6 weeks	Exercises in the clinic as a group exercise program	Pain: VAS	Both the clinic and home exercise programs were effective in decreasing pain levels in patients with knee osteoarthritis
Chen (2019)^23^ China	Diagnostic: NRS Inclusion: 60 years of age or older; experiencing knee pain on most days of the past month	HBEI: n = 71 (68.9 ± 7.78) CON: n = 70 (68.8 ± 6.96)	HBEI: 25.0 ± 3.45 CON: 25.4 ± 3.51	HBEI: 83% CON: 86%	Increase lower-limb muscle strength and balance, 30–40 min per day at least 3 days per week for 12 weeks	Health education booklet	Pain: WOMAC QoL: AIMS2-SF	HBEI and health education significantly reduced symptoms of KOA pain and quality of life, compared to an intervention that only involved health education

HBEI, home-based exercise intervention; CON, control group; OA, osteoarthritis; BMI, body mass index; HA, Hyaluronic Acid; ACR, American College of Rheumatology; JKOM, Japanese Knee Osteoarthritis Measure; IKHOAM, Ibadan Knee/Hip Osteoarthritis Outcome Measure; NRS, Numeric Rating Scale; VAS, visual analogue scale; WOMAC, Western Ontario and McMaster Universities Osteoarthritis Index; AQoL-2, Assessment of Quality of Life; KBA, kinesthesia balance and agility; RT, resistance training; PF, physical function; QoL, quality of life; SF36, Medical Outcomes Survey Short Form; AIMS2-SF, Arthritis Impact Measurement Scales 2 Short Form

### Methodological quality of the studies

Table 2 shows the methodological quality of the studies according to the PEDro scale: of the 12 included RCTs, 11 studies (92%) scored 6–8 points [15, 17–26], which indicates good methodological quality, but the other study (8%) scored 5 points and was considered of low methodological quality [16].

**Table 2:** Methodological classification assessed by PEDro scale

Authors	Eligibility criteria	Random allocation	Concealed allocation	Baseline comparability	Blind participants	Blind therapists	Blind assessor	Adequate follow-up dropout: < 15%	Intention-to-treat analysis	Between- group comparisons	Point estimates and variability	Score
O'Reilly (1999)^18^	Yes	Yes	Yes	Yes	No	No	No	Yes	Yes	Yes	Yes	7
Baker (2001)^15^	Yes	Yes	Yes	Yes	No	No	Yes	Yes	Yes	Yes	Yes	8
Thomas (2002)^19^	Yes	Yes	Yes	Yes	No	No	Yes	No	Yes	Yes	Yes	7
Brismee (2007)^17^	Yes	Yes	Yes	Yes	No	No	Yes	No	Yes	Yes	No	6
Doi (2008)^21^	Yes	Yes	Yes	Yes	No	No	No	Yes	Yes	Yes	Yes	7
Jenkinson (2009)^20^	Yes	Yes	Yes	Yes	No	No	No	No	Yes	Yes	Yes	6
Kawasaki (2009)^22^	Yes	Yes	Yes	Yes	No	No	No	Yes	No	Yes	Yes	6
Rogers (2012)^16^	Yes	Yes	Yes	No	No	No	No	Yes	No	Yes	Yes	5
Odole (2013)^24^	Yes	Yes	Yes	Yes	No	No	No	No	Yes	Yes	Yes	6
Bennell (2017)^25^	Yes	Yes	Yes	Yes	No	No	No	Yes	Yes	Yes	Yes	7
Colak (2017)^26^	Yes	Yes	Yes	Yes	No	No	No	No	Yes	Yes	Yes	6
Chen (2019)^23^	Yes	Yes	Yes	Yes	No	No	Yes	No	Yes	Yes	Yes	7

**Fig. 2:**
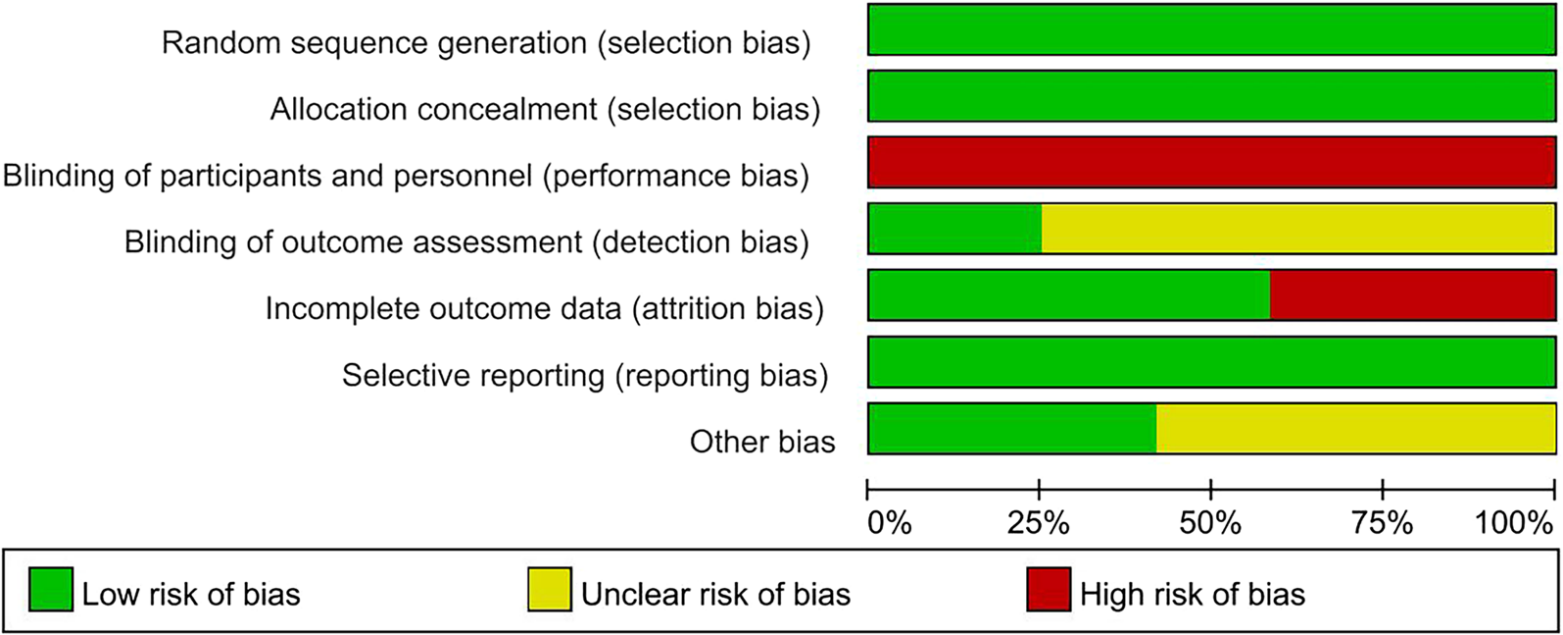
Risk of bias graph of included studies (Green, Low Risk of Bias; Yellow, Unclear Risk of Bias; Red, High Risk of Bias)

**Fig. 3:**
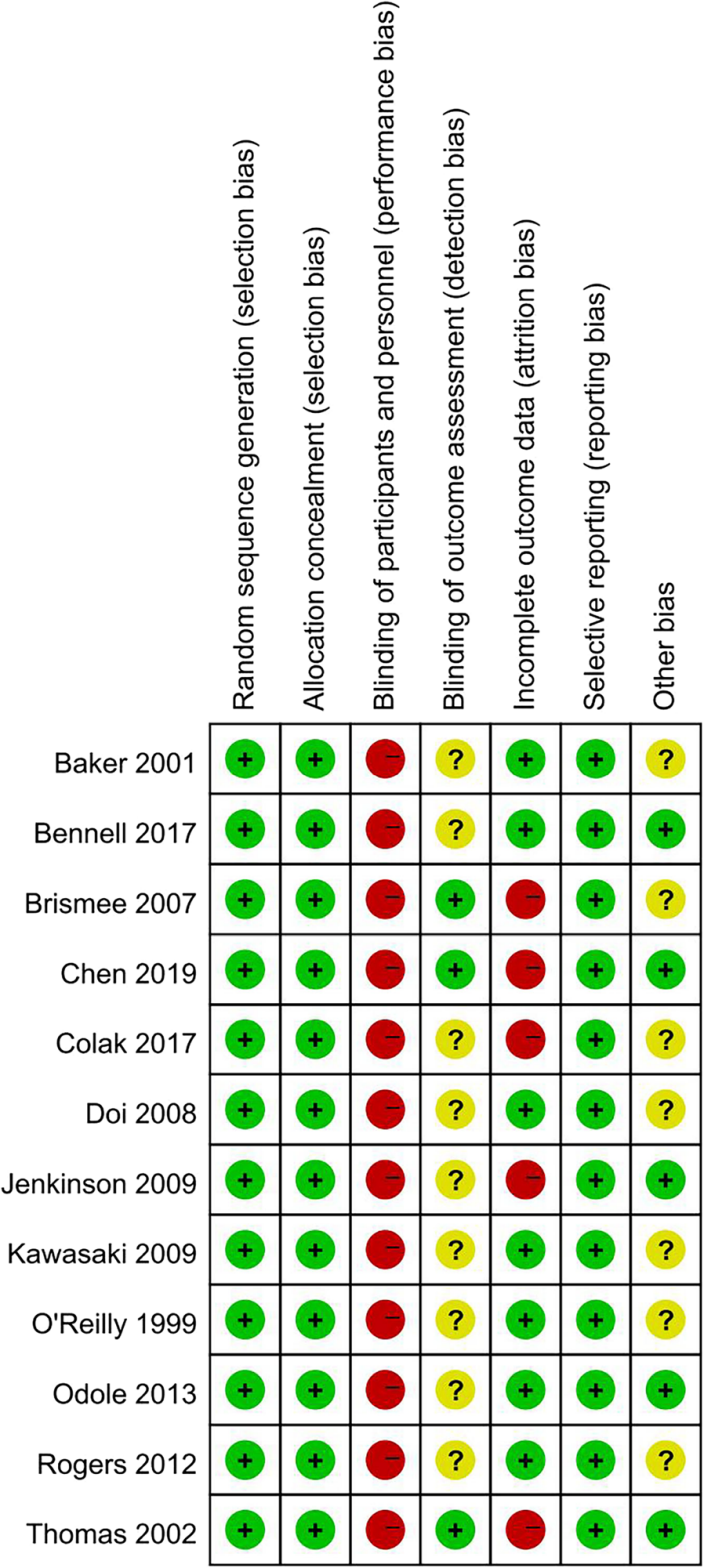
Risk of bias summary of included studies (Green, Low Risk of Bias; Yellow, Unclear Risk of Bias; Red, High Risk of Bias)

The detailed results of risk of bias are shown in Figs. 2 and 3. It is impossible to blind participants and therapists in all 12 studies (100%). Nine studies (75%) did not mention the blinding of outcome assessment, seven studies (58%) had other potential bias risks [15–18, 20–22, 24–26] and five studies (41%) had high dropout rates [17, 19, 20, 23, 26].

### Meta-analysis of outcomes

#### Effects of HBEI on pain

**Fig. 4:**
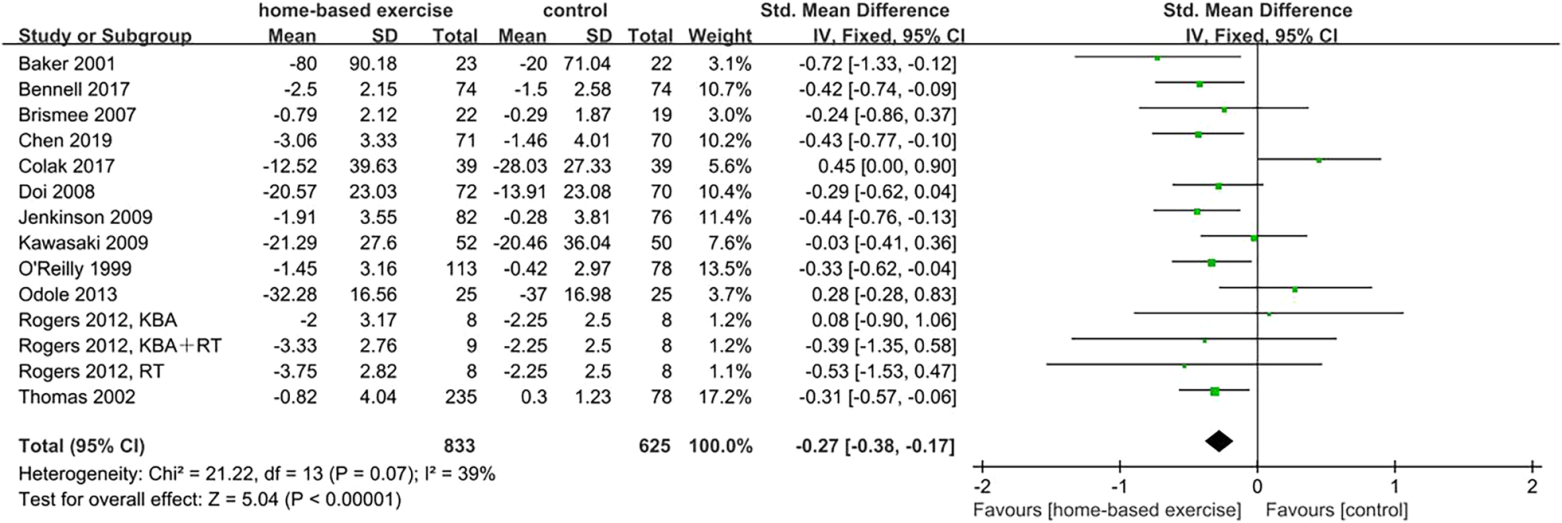
Effects of HBEI on pain

Twelve studies (100%) with 1442 participants evaluated the effect of HBEI on pain with KOA using the WOMAC pain subscale [15, 16, 18–20, 23], VAS [17, 21, 22, 24, 26], NRS [25]. Standard mean difference (SMD) and the fixed-effect model were used due to differences in the rating scale measurements and the low heterogeneity (*p* = 0.07, *I*^2^ = 39%), respectively. Analysis revealed that HBEI had a statistically significant effect on pain (SMD =  − 0.27, 95% CI [− 0.38, − 0.17], *p* < 0.001) (Fig. 4).

#### Effects of HBEI on physical function

**Fig. 5:**
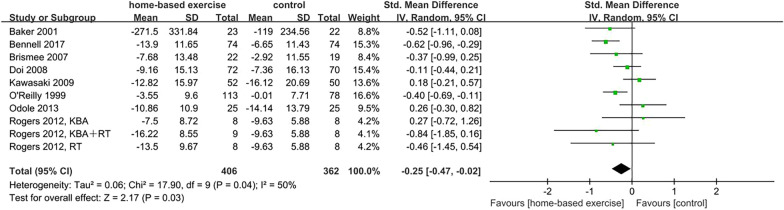
Effects of HBEI on physical function

Nine studies (75%) with 752 participants reported physical function with the WOMAC physical function subscale [15–18, 21, 25] the JKOM and the IKHOAM [22, 24]. Because of the use of different measurement scales and the existence of heterogeneity (*p* = 0.04, *I*^2^ = 50%), SMD and the random-effect model were applied. Home-based exercise interventions showed a significant improvement in physical function compared to the control group (SMD =  − 0.25, 95% CI [− 0.47, − 0.02], *p* = 0.03) (Fig. 5).

#### Effects of HBEI on quality of life

**Fig. 6:**
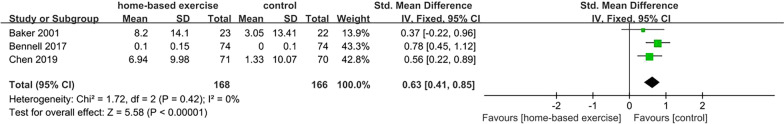
Effects of HBEI on quality of life

Three RCTs (25%) with 334 participants used the Medical Outcomes Survey Short Form (SF-36) [15], the AIMS2-SF [23] or the AQoL [25] as a measure of quality of life. Due to the use of different measurement scales and the low heterogeneity (*p* = 0.42, *I*^2^ = 0%), SMD and the fixed-effect model were used. The results indicated that HBEI had a significant impact on the quality of life of individuals with KOA compared to the control group (SMD = 0.63, 95% CI [0.41, 0.85], *p* < 0.001). However, subgroup analyses of the quality of life by control condition were not feasible, due to the content of the control group in all three studies included is health education and the low number of studies included in the meta-analysis (Fig. 6).

### Subgroup analysis by control condition

**Fig. 7:**
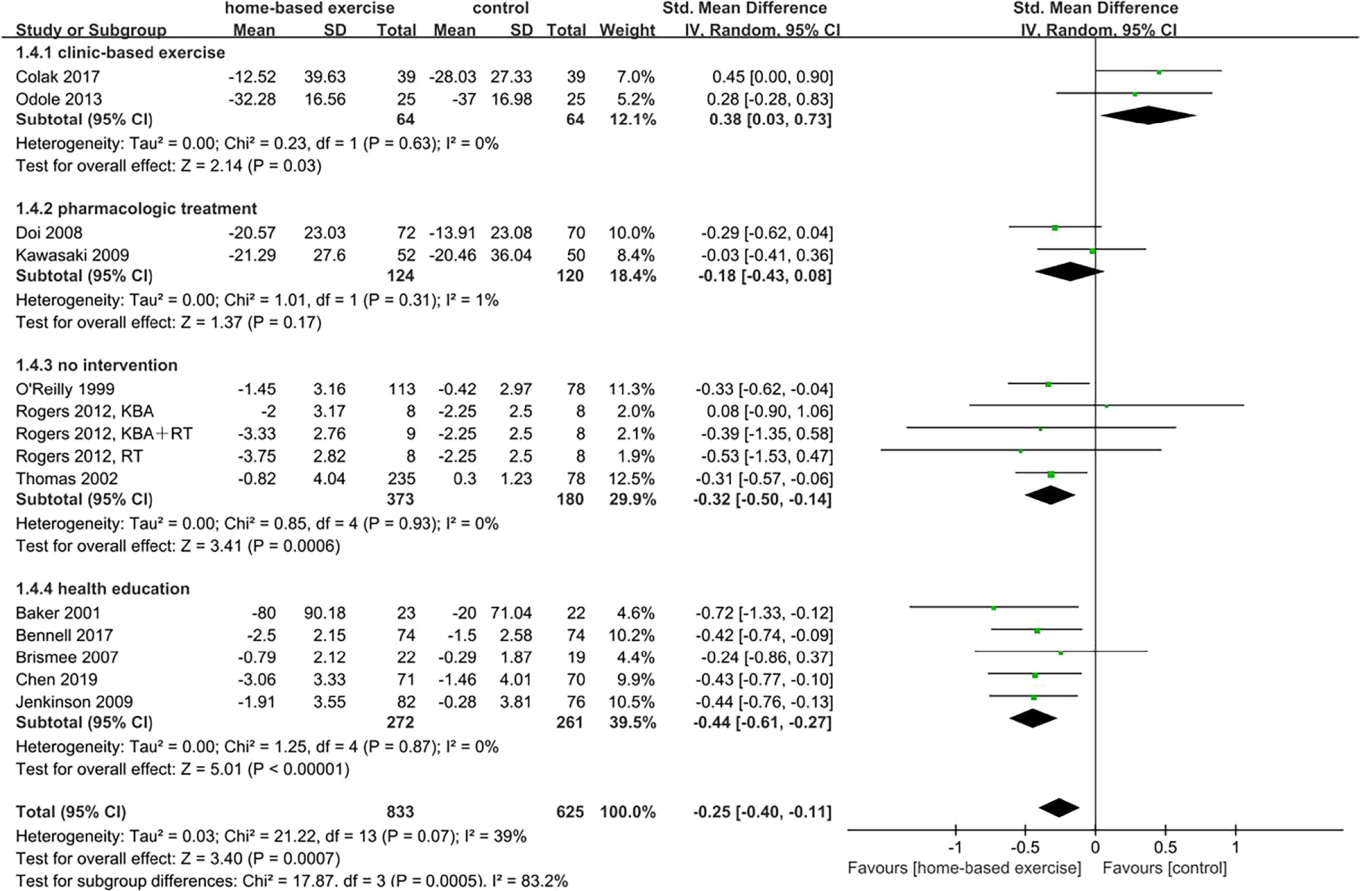
Effects of subgroup analysis of pain by control condition

**Fig. 8:**
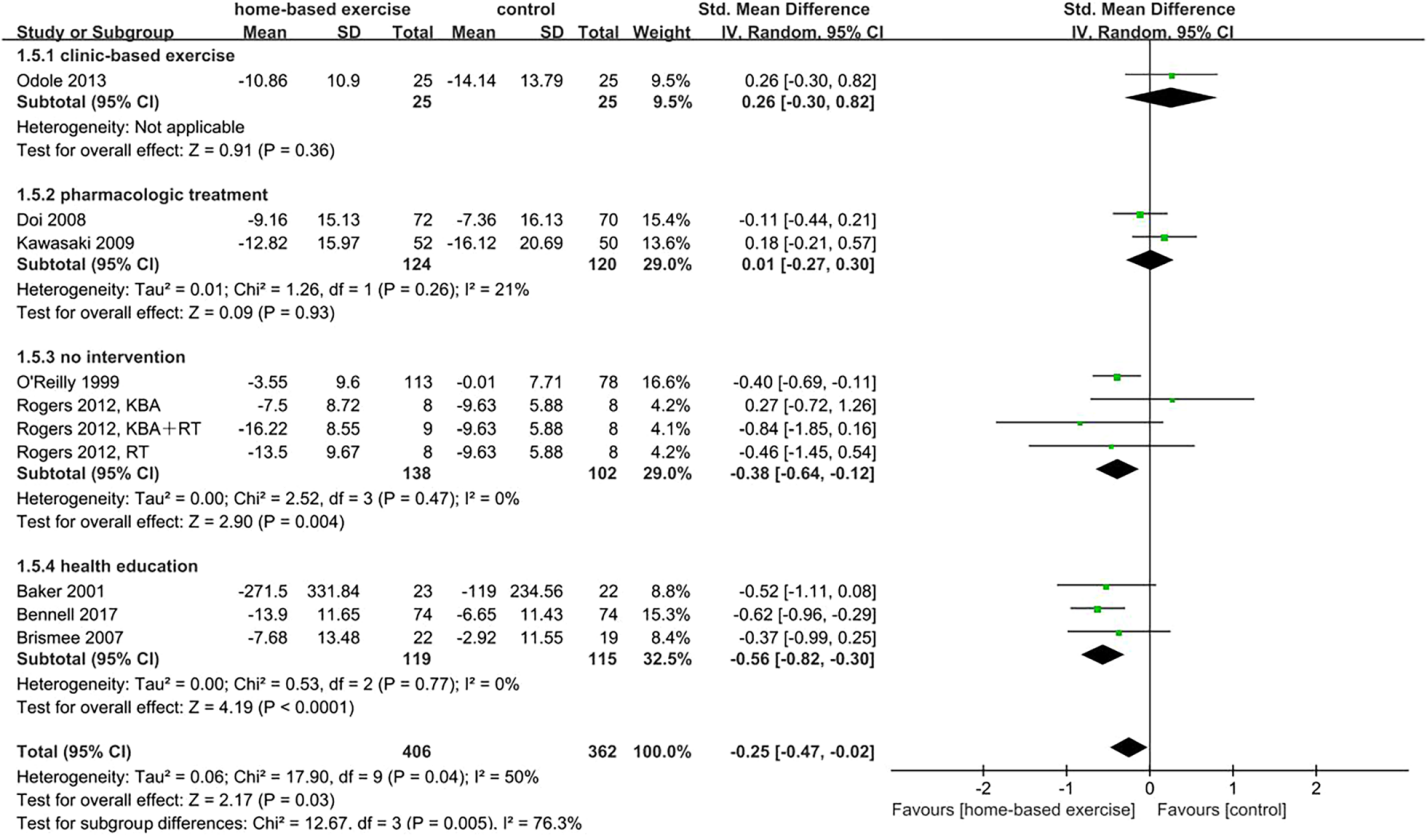
Effects of subgroup analysis of physical function by control condition

Studies were divided into four subgroups: (1) clinic-based exercise; (2) pharmacologic treatment, (3) no intervention; and (4) health education. With regard to the clinic-based exercise subgroup, there was significant relief of pain (SMD = 0.38, 95% CI [0.03, 0.73], *p* = 0.03) but there was no significant difference in physical function (SMD = 0.26, 95% CI [− 0.30, 0.82], *p* = 0.36) compared with the control group. In the subgroup with pharmacologic treatment, no significant difference was found in pain (SMD =  − 0.18, 95% CI [− 0.43, 0.08], *p* = 0.17) or physical function (SMD = 0.01, 95% CI [− 0.27, 0.30], *p* = 0.93). Within no intervention as the control group, the effects of HBEI on pain (SMD =  − 0.32, 95% CI [− 0.50, − 0.14], *p* = 0.0006) and physical function (SMD =  − 0.38, 95% CI [− 0.64, − 0.12], *p* = 0.004) were significant. The health education subgroup showed that participants in the HBEI group had significantly less pain (SMD =  − 0.44, 95% CI [− 0.61, 0.27], *p* < 0.001) and significantly better physical function (SMD =  − 0.56, 95% CI [− 0.82, − 0.30], *p* < 0.001). Subgroup interaction was significant for pain (*p* = 0.0005) and physical function (*p* = 0.005) (Figs. 7 and 8).

### Publication bias

**Fig. 9:**
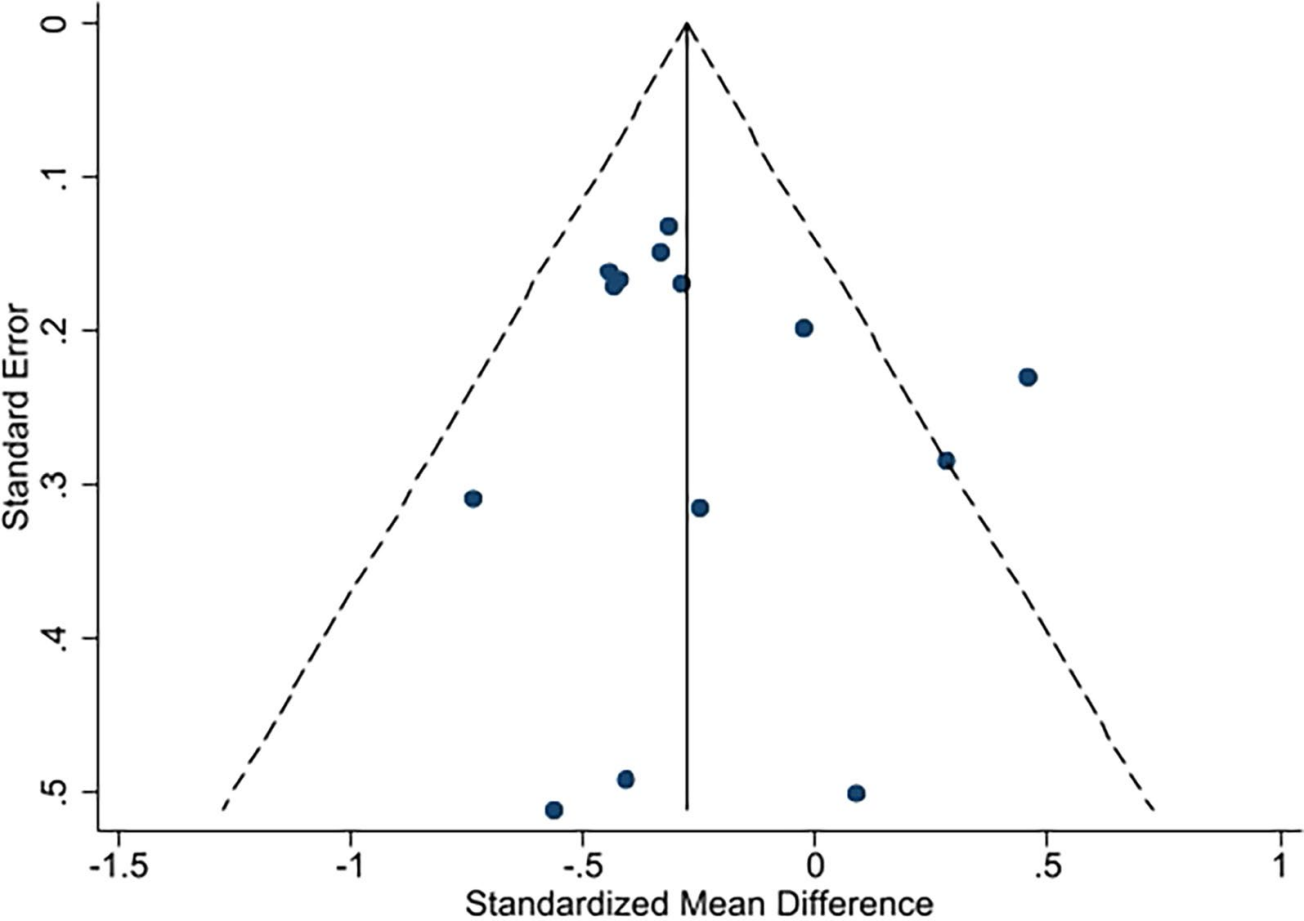
Funnel plot of pain

**Fig. 10:**
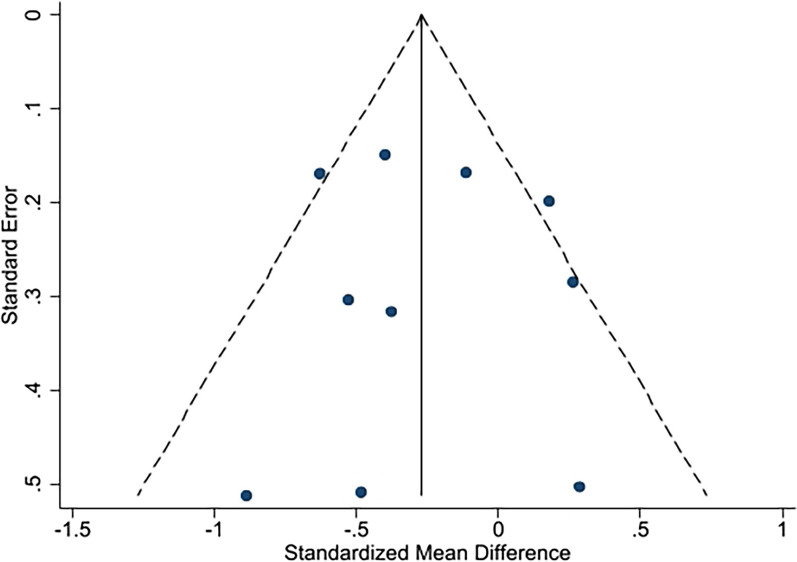
Funnel plot of physical function

The results were displayed through a funnel plot and Eggar's test. The test found funnel plot symmetry, indicating that there was no significant evidence of publication bias for pain (*p* = 0.489; Fig. 9) or physical function (*p* = 0.872; Fig. 10).

## Discussion

### Summary of main findings

The results showed that HBEI could significantly alleviate pain and improve physical function and quality of life. Furthermore, subgroup analyses revealed that the control condition influenced the assessment of intervention effects. The validity of the present meta-analysis may be low due to the heterogeneity in the design and choice of outcomes of the included studies, which should be interpreted cautiously.

Knee pain can lead to many problems, such as physical disability, poor quality of life and socioeconomic burden [27]. Our analysis found that HBEI could significantly improve pain, and this result was similar to that of Anwer et al. [11]. Musculoskeletal conditions may increase the risk of chronic disease [28] but HBEI has been proven to have good therapeutic effects on a variety of diseases (e.g. stroke, Parkinson’s, skeletal muscle atrophy, diabetes mellitus) [29–32]. Therefore, home exercise may improve some complications of KOA while relieving pain. However, a greater understanding of why musculoskeletal conditions may increase the risk of chronic disease is needed [28] and the effect of home exercise on the complications of KOA needs further investigation. Juhl's review found that an exercise frequency of at least three times per week was more effective in alleviating KOA pain and reducing the rate of disability than an exercise frequency of at least twice a week [33] which is similar to the American College of Sports Medicine (ACSM) recommended routine. Furthermore, multiple forms of exercise have been found that could reduce pain, such as Tai Chi, aerobics and strength training. Therefore, the individual can choose the appropriate programmes of home exercise with minimal resources according to their own condition and the doctor's advice to relieve pain.

With regard to the effect of HBEI on physical function, previous reviews have reported significant improvement compared with the control group [11, 12], and similar results were reported in this study. Among the high-quality studies included here, most tended to select muscle strengthening or stretching exercises that targeted the knee joint only [15, 16, 18–23, 25, 26]. However, when performing physical activity, the lower extremities form a whole kinematic chain, making it impossible for the hip, knee or ankle joints to work entirely independently and, instead, they may affect each other [34]. Therefore, it is postulated that to achieve improvements in physical function, it may be necessary to integrate other joint exercises (e.g. hip) in the intervention [35]. In addition, a small but growing number of studies have shown the effects of traditional Chinese exercise (e.g. Tai Chi and Wuqinxi) on the physical function of KOA individuals [36–38].

With regard to the effect of HBEI on individuals' quality of life, in the present review the unsupervised HBEI showed a significant effect compared with the health education control, as has been found in some previous reviews [39, 40]. Furthermore, as a cost-effective non-pharmacological intervention, health education has always been recommended in KOA management but should be combined with exercise therapy and not provided as a stand-alone treatment [41]. Three studies included in the meta-analysis effectively improved participants' pain or physical function. Pain relief and better physical function may provide more convenience and greater range of motion for participants in daily life, which improves quality of life to some extent. In addition, to achieve a better quality of life, it may be necessary to combine HBEI with cognitive behavioural therapies [40]. However, due to the limited number of included studies there may be heterogeneity, thus future studies need to consider including a sufficiently large number of RCTs and dividing subgroup by control condition to examine the effect of HBEI on quality of life.

We noted that HBEI improved mental health in the included RCTs [18, 20]. Individuals with KOA are at elevated risk for psychological distress [42, 43], especially during the COVID-19 pandemic. Mental health (e.g. anxiety and depression) is not only related to an individual's quality of life but is also an important social issue. Therefore, it is our future research plan to explore the effect of HBEI on the mental health of KOA individuals.

### Subgroup analyses

The effect of exercise therapy for KOA varies significantly depending on the different control groups [44]. In contrast a previous meta-analysis [11], more detailed subgroup division was conducted in this study. To accurately estimate the effect of HBEI, we performed subgroup analyses on pain and physical function according to the control conditions.

Compared with the clinic-based exercise subgroup, the results of supervised HBEI showed similar physical function improvement but a significant relief of pain. This is consistent with previous studies that have summarized the role of exercise in the management of KOA, producing evidence-based recommendations that clinical exercise and home exercise are equally effective [45, 46]. The pain-relieving advantages of home exercise therapy may be due to providing a comforting atmosphere, thus reducing the psychological stress of the participants and thereby inducing active participation of the family and caregivers. In addition, HBEI can reduce the costs and time needed to travel to a rehabilitation centre [30, 47]. With regard to the pharmacologic treatment subgroup, similar effects of unsupervised HBEI and pharmacological treatment were found in terms of pain relief and improvement in physical function. Some previous reviews demonstrated similar effects of HBEI on pain and functional improvement compared with non-steroidal anti-inflammatory drugs or intra-articular hyaluronic acid [39, 48]. As for the subgroup with no intervention, HBEI based on muscle strengthening was used in all three RCTs included (two unsupervised and one supervised) [16, 18, 19]. For the health education subgroup, unsupervised HBEI was used in all five RCTs included (four muscle-strengthening interventions and one Tai Chi intervention) [15, 17, 20, 23, 25]. The results showed that the improvements in pain and physical function with HBEI were significant compared with no intervention and health education. This may be due to the fact that moderate exercise can enhance muscle mass [49], promote intra-joint material exchange and blood circulation, reduce the accumulation of inflammatory factors, maintain the biomechanical balance of joint structure and mitigate joint load and cartilage damage, which in turn relieves pain [50, 51]. On the other hand, exercise therapy increases lower limb strength and range of motion, and also protects patellar cartilage composition to a limited extent, which in turn improves physical function [52–54]. Resistance, aerobic exercise and flexibility are the most common training modalities for KOA management, but studies of multidimensional exercise interventions based on Tai Chi may provide a broader range of recommendations for future HBEI studies [34]. However, with regard to physical function, we noted that only one RCT was included in the clinic-based exercise subgroup. This small sample size may be affected by chance and the reliability of this result needs to be further explored.

### Implications

Even though there are some limitations to this review, there are also some implications for practice. HBEI can provide effective pain management for individuals with clinical rehabilitation limitations and finds advantage in improving the physical function and quality of life of KOA individuals. Due to the included studies using interventions with different frequencies of exercise, subgroup analysis based on duration was not possible. Therefore, researchers need to develop uniform clinical norms to help explore the effect of duration of HBEI on the management of KOA. In addition, growing evidence supports the effectiveness of traditional Chinese exercise [55–57] and taking it as an HBEI may lead to greater improvements for KOA individuals. However, given the fact that many KOA individuals have more than one chronic condition, Tai Chi may be a better choice of intervention [56].

Our study showed that unsupervised HBEI improved the quality of life compared with health education. The effect of other forms of exercise and in combination with cognitive behavioural therapies on the quality of life remains to be further explored. Supervised HBEI provided similar pain relief and improved physical function compared to clinic-based exercise treatment, with the Internet and smartphone applications providing more effective options for the supervision of physical therapists and the implementation of HBEI [58]. Notably, although HBEI can be used as a cost-effective and convenient exercise therapy, the acceptable intensity and duration of the target population still need to be considered in practical applications.

### Limitations

First, the generalizability of this meta-analysis is moderately limited, in that our results are only applicable to KOA individuals who do not choose knee replacement. Second, only studies published in English were included due to the lack of reviewers who were fluent in other languages. Third, the literature search was not comprehensive enough and the large heterogeneity in the included RCTs (such as frequency, intensity, duration) may have affected the effectiveness of the aggregated results. Finally, most of the included studies used strengthening exercises, so there was a lack of analysis on other types of exercise such as aerobics, blood flow restriction training, balance and proprioceptive training.

## Conclusions

HBEI is a promising strategy for KOA management when clinical treatment conditions are limited. The results provided evidence of a favourable effect or tendency of HBEI on improving the knee pain, physical function and quality of life. Additionally, the meta-analyses showed the favourable effects of HBEI versus no intervention and health education and were able to achieve the effects of clinic-based exercise and pharmacological treatment. As an important supplement to clinical treatment, HBEI can provide greater benefit to individuals with KOA, but individual disease complications and the dosimetry of the exercise need to be considered in practice.

## Supplementary Information

**Additional file 1.** Details of the literature search.

## Data Availability

The present study was a meta-analysis of previous published studies.

## Abbreviations

KOA: Knee osteoarthritis
RCT: Randomized controlled trials
PEDro: The Physiotherapy Evidence Database
RevMan: Review Manager
PRISMA: Preferred Reporting Items For Systematic Reviews And Meta-Analyses
MeSH: Medical Subject Headings
WOMAC: Western Ontario and McMaster Universities Osteoarthritis Index
VAS: Visual Analogue Scale
NRS: Numerical Rating Scale
JKOM: Japanese Knee Osteoarthritis Measure
IKHOAM: Ibadan Knee/Hip Osteoarthritis Outcome Measure
SF-36: Medical Outcomes Survey Short Form
AQoL: Assessment of Quality of Life
AIMS2-SF: Arthritis Impact Measurement Scales 2 Short Form
HBEI: Home-based exercise interventions
