# Supplementary material for: Effectiveness of home-based exercise interventions on pain, physical function and quality of life in individuals with knee osteoarthritis: a systematic review and meta-analysis
Source: J Orthop Surg Res. 2023 Jul 17;18:503. doi: 10.1186/s13018-023-04004-z (PMC10351144; doi:10.1186/s13018-023-04004-z)
Supplement: Supplementary file 1 — Additional file 1. Details of the literature search. [file 13018_2023_4004_MOESM1_ESM.docx]

**Appendix 1.** Details of the literature search and the number of citations found in each database

| **PubMed (from the inception to August 2, 2022)** | | |
| --- | --- | --- |
| Search number | Search Details | Results (N) |
| #1 | "osteoarthritis, knee"[MeSH Terms] OR "knee osteoarthritis"[Title/Abstract] OR "KOA"[Title/Abstract] OR "knee OA"[Title/Abstract] | 30,448 |
| #2 | ("knee"[Title/Abstract] OR "knee joint"[Title/Abstract]) AND ("arthrosis"[Title/Abstract] OR "arthritis"[Title/Abstract] OR "osteoarthr*"[Title/Abstract] OR "OA"[Title/Abstract] OR "degeneration"[Title/Abstract] OR "degenerative"[Title/Abstract]) | 46,816 |
| #3 | "arthritis, rheumatoid"[MeSH Terms] OR "rheumatoid arthritis"[Title/Abstract] | 160,562 |
| #4 | (#1 OR #2) NOT #3 | 48,615 |
| #5 | "home-based" or "home" or "base" or "based" | 4,947,103 |
| #6 | ("Exercise"[Mesh]) OR (Exercise[Title/Abstract]) OR (Exercises[Title/Abstract]) OR (Physical Activity[Title/Abstract]) OR (Activities, Physical[Title/Abstract]) OR (Activity, Physical[Title/Abstract]) OR (Physical Activities[Title/Abstract]) OR (Exercise, Physical[Title/Abstract]) OR (Exercises, Physical[Title/Abstract]) OR (Physical Exercise*[Title/Abstract]) OR (Acute Exercise*[Title/Abstract]) OR (Exercise*, Acute[Title/Abstract]) OR (Exercise*, Isometric[Title/Abstract]) OR (Isometric Exercise*[Title/Abstract]) OR (Exercise, Aerobic[Title/Abstract]) OR (Aerobic Exercise*[Title/Abstract]) OR (Exercises, Aerobic[Title/Abstract]) OR (Exercise Training*[Title/Abstract]) OR (Training*, Exercise[Title/Abstract]) | 527,260 |
| #7 | #5 and #6 | 111,259 |
| #8 | "animal"[Title/Abstract] OR "animals"[Title/Abstract] OR "arthroplast*"[Title/Abstract] | 1,288,762 |
| #9 | (#4 AND #7) NOT #8 | 1,282 |

| **Web of Science Core Collection (from the inception to August 2, 2022)** | | |
| --- | --- | --- |
| Search number | Search Details | Results(N) |
| #1 | TS=("knee osteoarthritis" OR "KOA" OR "knee OA") | 29322 |
| #2 | (TS=((knee OR "knee joint"))) AND TS=((arthrosis OR arthritis OR osteoarthr* OR "OA" OR degeneration OR degenerative)) |  |
| #3 | TS=("rheumatoid arthritis") |  |
| #4 | (#1 OR #2) NOT #3 |  |
| #5 | TS=("home-based" OR "home" OR "base" OR "based") |  |
| #6 | TS=(("Exercise") OR (Exercise) OR (Exercises) OR (Physical Activity) OR (Activities, Physical) OR (Activity, Physical) OR (Physical Activities) OR (Exercise, Physical) OR (Exercises, Physical) OR (Physical Exercise*) OR (Acute Exercise*) OR (Exercise*, Acute) OR (Exercise*, Isometric) OR (Isometric Exercise*) OR (Exercise, Aerobic) OR (Aerobic Exercise*) OR (Exercises, Aerobic) OR (Exercise Training*) OR (Training*, Exercise)) | 866138 |
| #7 | #5 and #6 |  |
| #8 | TS=("animal" OR "animals" OR "arthroplast*") | 1449632 |
| #9 | (#4 and #7) NOT #8 | 1998 |

| **Embase (from the inception to August 2, 2022)** | | |
| --- | --- | --- |
| Search number | Search Details | Results(N) |
| #1 | 'knee osteoarthritis'/exp OR 'knee osteoarthritis':ab,ti OR 'koa':ab,ti OR 'knee oa':ab,ti | 45397 |
| #2 | ('knee':ab,ti OR 'knee joint':ab,ti) AND ('arthritis':ab,ti OR 'osteoarthr*':ab,ti OR 'oa':ab,ti OR 'degeneration':ab,ti OR 'degenerative':ab,ti OR 'arthralgia':ab,ti) | 68347 |
| #3 | 'rheumatoid arthritis'/exp OR 'rheumatoid arthritis':ab,ti | 262738 |
| #4 | #1 OR #2 |  |
| #5 | #4 NOT #3 | 69160 |
| #6 | 'exercise':ab,ti OR exercise:ab,ti OR exercises:ab,ti OR 'physical activity':ab,ti OR 'activities, physical':ab,ti OR 'activity, physical':ab,ti OR 'physical activities':ab,ti OR 'exercise, physical':ab,ti OR 'exercises, physical':ab,ti OR 'physical exercise*':ab,ti OR 'acute exercise*':ab,ti OR 'exercise*, acute':ab,ti OR 'exercise*, isometric':ab,ti OR 'isometric exercise*':ab,ti OR 'exercise, aerobic':ab,ti OR 'aerobic exercise*':ab,ti OR 'exercises, aerobic':ab,ti OR 'exercise training*':ab,ti OR 'training*, exercise':ab,ti | 578561 |
| #7 | 'home-based':ab,ti OR 'home':ab,ti OR 'base':ab,ti OR 'based':ab,ti | 5639576 |
| #8 | #6 AND #7 | 130701 |
| #9 | 'animal*':ab,ti OR 'arthroplast*':ab,ti | 1561029 |
| #10 | #5 AND #8 NOT #9 | 2042 |

| **Cochrane Library (from the inception to August 2, 2022)** | | |
| --- | --- | --- |
| Search number | Search Details | Results(N) |
| #1 | MeSH descriptor: [Osteoarthritis, Knee] explode all trees | 5183 |
| #2 | ("knee osteoarthritis"):ti,ab,kw OR ("KOA"):ti,ab,kw OR ("knee OA"):ti,ab,kw | 8909 |
| #3 | (knee or "knee joint"):ti,ab,kw AND (arthrosis or arthritis or osteoarthr* or "OA" or degeneration or degenerative):ti,ab,kw | 15528 |
| #4 | #1 OR #2 OR #3 | 15542 |
| #5 | MeSH descriptor: [Arthritis, Rheumatoid] explode all trees | 6513 |
| #6 | (rheumatoid arthritis):ti,ab,kw | 17603 |
| #7 | #5 OR #6 | 17927 |
| #8 | #4 NOT #7 | 14681 |
| #9 | home-based':ab,ti OR 'home':ab,ti OR 'base':ab,ti OR 'based':ab,ti | 324352 |
| #10 | MeSH descriptor: [Exercise] explode all trees | 28634 |
| #11 | (("Exercise") OR (Exercise) OR (Exercises) OR (Physical Activity) OR (Activities, Physical) OR (Activity, Physical) OR (Physical Activities) OR (Exercise, Physical) OR (Exercises, Physical) OR (Physical Exercise*) OR (Acute Exercise*) OR (Exercise*, Acute) OR (Exercise*, Isometric) OR (Isometric Exercise*) OR (Exercise, Aerobic) OR (Aerobic Exercise*) OR (Exercises, Aerobic) OR (Exercise Training*) OR (Training*, Exercise)):ti,ab,kw (Word variations have been searched) | 156130 |
| #12 | (#10 OR #11) and #9 | 45218 |
| #13 | (animal OR animals OR arthroplast*):ti,ab,kw | 46725 |
| #14 | #8 AND #12 NOT #13 | 1226 |
| #15 | #14 with the use of the ‘Trials’ filter in the Cochrane Library | 1208 |

| **CINAHL (from the inception to August 2, 2022)** | | |
| --- | --- | --- |
| Search number | Search Details | Results(N) |
| S1 | (MH "Osteoarthritis, Knee") | 14,335 |
| S2 | (MH "Osteoarthritis, Knee") OR ("knee osteoarthritis" OR "KOA" OR "knee OA" ) | 27,365 |
| S3 | ( knee OR "knee joint" ) AND (arthrosis OR arthritis OR osteoarthr* OR "OA" OR degeneration OR degenerative ) | 27,350 |
| S4 | (MH "Arthritis, Rheumatoid+") | 30,807 |
| S5 | (MH "Arthritis, Rheumatoid+") OR "rheumatoid arthritis" | 39,240 |
| S6 | home-based or "home" or "base" or "based" | 1,086,784 |
| S7 | (MH "Exercise+") | 126,442 |
| S8 | (MH "Exercise+") or ("Exercise" OR Exercise OR Exercises OR Physical Activity OR Activities, Physical OR Activity, Physical OR Physical Activities OR Exercise, Physical OR Exercises, Physical OR Physical Exercise* OR Acute Exercise* OR Exercise*, Acute OR Exercise*, Isometric OR Isometric Exercise* OR Exercise, Aerobic OR Aerobic Exercise* OR Exercises, Aerobic OR Exercise Training* OR Training*, Exercise) | 297,625 |
| S9 | animal OR animals OR arthroplast* | 321,812 |
| S10 | (S2 OR S3) NOT S5 | 33,600 |
| S11 | (S10 AND S6 AND S8) NOT S9 | 1,101 |
